# Wheat-Net: An Automatic Dense Wheat Spike Segmentation Method Based on an Optimized Hybrid Task Cascade Model

**DOI:** 10.3389/fpls.2022.834938

**Published:** 2022-02-10

**Authors:** Jiajing Zhang, An Min, Brian J. Steffenson, Wen-Hao Su, Cory D. Hirsch, James Anderson, Jian Wei, Qin Ma, Ce Yang

**Affiliations:** ^1^College of Information and Electrical Engineering, China Agricultural University, Beijing, China; ^2^The State Key Laboratory of Management and Control for Complex Systems, Institute of Automation, Chinese Academy of Sciences, Beijing, China; ^3^School of Artificial Intelligence, University of Chinese Academy of Sciences, Beijing, China; ^4^Department of Bioproducts and Biosystems Engineering, University of Minnesota, Saint Paul, MN, United States; ^5^Department of Plant Pathology, University of Minnesota, Saint Paul, MN, United States; ^6^College of Engineering, China Agricultural University, Beijing, China; ^7^Department of Agronomy and Plant Genetics, University of Minnesota, Saint Paul, MN, United States

**Keywords:** wheat spike, instance segmentation, Hybrid Task Cascade model, challenging dataset, non-structural field

## Abstract

Precise segmentation of wheat spikes from a complex background is necessary for obtaining image-based phenotypic information of wheat traits such as yield estimation and spike morphology. A new instance segmentation method based on a Hybrid Task Cascade model was proposed to solve the wheat spike detection problem with improved detection results. In this study, wheat images were collected from fields where the environment varied both spatially and temporally. Res2Net50 was adopted as a backbone network, combined with multi-scale training, deformable convolutional networks, and Generic ROI Extractor for rich feature learning. The proposed methods were trained and validated, and the average precision (AP) obtained for the bounding box and mask was 0.904 and 0.907, respectively, and the accuracy for wheat spike counting was 99.29%. Comprehensive empirical analyses revealed that our method (Wheat-Net) performed well on challenging field-based datasets with mixed qualities, particularly those with various backgrounds and wheat spike adjacence/occlusion. These results provide evidence for dense wheat spike detection capabilities with masking, which is useful for not only wheat yield estimation but also spike morphology assessments.

## Introduction

Wheat is the most widely cultivated cereal crop and also one of the most important food sources for humans in the world. The spike is the most important component of the wheat plant because it contains the seeds that are harvested and ultimately consumed. Therefore, in-field automated wheat spike detection based on remote sensing is an important step toward yield estimation and spike morphology assessments.

To detect wheat spikes, the remote sensing imaging devices are useful tools to replace traditional artificial detection (Aparicio et al., 2000). Hyperspectral imaging cameras can provide rich spectral information for wheat detection (Shen et al., 2021), but the cost of hyperspectral imaging is expensive, which restricts the application in various fields (Zhang et al., 2020). Thus, the cheaper RGB imaging camera is a realistic alternative to achieve effective wheat detection. Deep learning (DL) with strong feature learning abilities has spawned a multitude of applications in RGB images. It encodes the composition of lower-level features into more discriminative higher-level features (Ni et al., 2019). DL can solve more complex problems with higher precision and has been successfully used in plant classification (Sun et al., 2019; Yang et al., 2019; Khaki et al., 2020), yield prediction (Pound et al., 2017; de Luna et al., 2020; Zhuang et al., 2020), growth monitoring (Kovalchuk et al., 2017; Qiongyan et al., 2017), and disease/pest detection (Senthilkumar et al., 2017; da Silva et al., 2019; Desai et al., 2019). Thus, DL, with its advantages of high precision and intelligence, is an attractive alternative to conventional wheat spike detection methods (Germain et al., 1995; Cointault et al., 2008a,b).

Recently, DL has been shown to perform well in a wide variety of wheat spike detection studies. Some previous works involving wheat detection have been conducted under laboratory conditions and controlled environments (Hasan et al., 2018; Sadeghi-Tehran et al., 2019; Chandra et al., 2020; Misra et al., 2020). Laboratory-based experiments have good lighting conditions and a clean background, which is not the case for field-based research, which is more complicated and yields images where the background usually contains a lot of disturbances (including soil and weeds). The complicated background greatly increases the difficulty of resolving individual wheat spikes but represents the actual growing environment of wheat. Thus, models developed from the field are more realistic of real-world conditions for wheat cultivation. Several in-field spike detection and counting studies have been conducted (David et al., 2020, 2021; Xu et al., 2020; Wang et al., 2021). Among them, David et al. (2021) constructed a more diverse and less noisy Global Wheat Head Detection (GWHD) dataset, which promoted the development of wheat spike detection. The detection results from these studies were based on bounding boxes, which can be used for counting wheat spikes. However, the precise pixel areas of wheat are often required in wheat management (such as evaluation of spikes disease and accurate yield prediction), which cannot be achieved by detecting the bounding box only by segmentation. Segmentation provides information such as size, shape, and relative location of the segments in the image, which can be used for phenotypic traits such as spike size, shape, distribution, and wheat yield potential. Therefore, it is necessary to explore an approach of segmenting wheat spikes to meet the needs for precise spike areas in wheat management.

There are some researchers who have used semantic segmentation algorithms to segment wheat spikes in the field with a simpler environment by controlling some factors in the experiment. For example, in implementing a Fully Convolutional Network (FCN) segmentation model of individual wheat spikes, Zhang et al. (2019) positioned spikes to avoid occlusion–an intervention that does not simulate the actual growing conditions of wheat in the field. Alkhudaydi et al. (2019) employed FCN to segment multiple wheat spikes, which achieved a Mean Accuracy (MA) of classification of > 76%. However, their model performed poorly under challenging conditions caused by variable lighting and weather (Alkhudaydi et al., 2019). Tan et al. (2020) performed simple linear iterative clustering (SLIC) for superpixel segmentation of digital wheat images, which resulted in a high accuracy (94.01%) under high nitrogen fertilizer level and a lower accuracy (80.8%) under no nitrogen fertilizer application. Ma et al. (2020) developed EarSegNet to segment multiple wheat spikes from canopy images captured under field conditions and realized a precision of 79.41%. However, semantic segmentation algorithms cannot segment wheat spikes out individually when they are obstructed by other spikes, which is a common situation under field conditions.

Instance segmentation can effectively segment partially obstructed wheat spikes. This method localizes objects of interest in an image at the pixel level, which achieves both object detection and semantic segmentation (Li et al., 2017; Chen et al., 2019). With instance segmentation, the segmented objects are generally represented by masks and a bounding box (bbox); however, few studies have been advanced using instance segmentation for detecting wheat spikes under field settings. Qiu et al. (2019) used a Mask RCNN model to reliably detect wheat spikes (mean average precision is 0.9201) with different shapes and features in the field. However, to achieve these results, they used a background plate to block complex backgrounds and also a shade shed to provide even lighting, which reduced the complexities of image capture and subsequent annotation. They also divided the original image into many smaller images, which resulted in image distortion. This, in turn, resulted in images with only partial objects or no objects at all, which would destroy the integrity of the wheat spikes. In our previous research (Su et al., 2021), we basically realized the instance segmentation of wheat in a complex field environment, but its low accuracy cannot meet the needs of practical applications and further research is necessary to achieve high-precision instance segmentation.

In summary, the object detection of wheat is insufficient for accurate phenotype study and semantic segmentation cannot segment common occlusive wheat spikes. Instance segmentation methods can solve the above problems, but the conventional instance segmentation methods of wheat are either in laboratory conditions or controlled environments or have low accuracy, etc., which may not be suitable for phenotyping spikes under complex field environment. Therefore, it is necessary to explore a more applicable and accurate approach for segmenting wheat spikes under field conditions. Therefore, the specific objectives of this study were to: construct a new instance segmentation model (called Wheat-Net) based on a multi-task Hybrid Task Cascade (HTC) model (Chen et al., 2019) that can precisely instance segment wheat spikes in high densities in the field. Comprehensive empirical analyses reveal that Wheat-Net achieved excellent performance on a challenging dataset with various complex backgrounds and a high level of obstruction. In a complex, unstructured environment, our method not only accurately detected the wheat spikes with bounding boxes but also extracted spike regions from the background at the pixel level.

## Materials and Methods

### Data Collection

Wheat genotypes were sown in field plots on the St. Paul campus of the University of Minnesota (UMN) in 2019. These genotypes included mostly breeding lines from the UMN hard red spring wheat breeding program, which can vary for different spike morphology traits such as color, shape types as well as spike density. The images at the late flowering stage (July 11) to the milk stage of maturity (August 2) were collected from the field including 20 wheat genotypes, which can enhance the adaptability of the model to different wheat genotypes. In the complex field environment, we used the camera of Canon EOS Rebel T7i (autofocus single-lens reflex, pixels: 6,000 × 4,000) to collect image data under different weather conditions (including sunny and cloudy days). The exposure time, white balance, and ISO speed were automatically set based on the automatic mode of camera. The distance from the object is about 1–2 m. The wheat images collected had complex backgrounds, including weeds, soil, blurred wheat, blue sky, and white clouds. We expect that users take images of the wheat/barley trial plots with very loose image acquisition requirements (e.g., imaging angle and distance). Therefore, we acquired the current dataset with various angles and distances, which also can increase the diversity of data and enhance the adaptability and robustness of the model.

**FIGURE 1:**
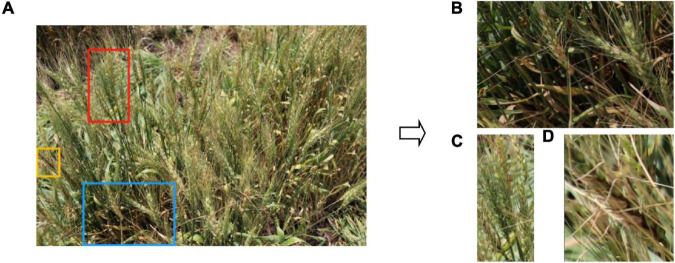
**(A)** An example of an original image of a wheat plot indicating sections (blue, red, and yellow boxes) enlarged to show **(B)** an area with incomplete illumination, **(C)** adjacent spikes in close proximity, and **(D)** partial spikes on the edge of the images.

**FIGURE 2:**
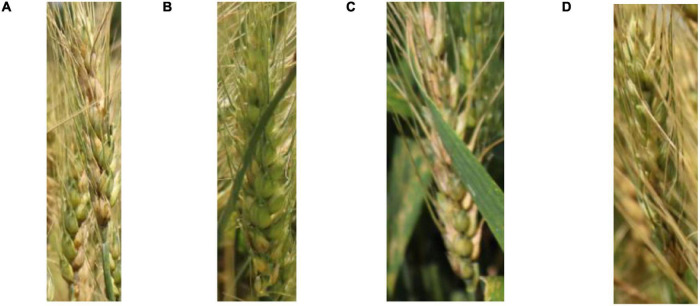
Examples of various spike occlusion scenarios: **(A)** spike over spike, **(B)** leaf over spike, **(C)** stem over spike, and **(D)** awns over spike.

Wheat is typically a dense crop and the images (Figure 1A) collected contained as many as 124 spikes per image. In addition, it was common that portions of images had insufficient illumination (blue box with zoom-in shown in Figure 1B). Moreover, the above factors also resulted in many problems such as spike adjacence (Red box with zoom-in shown in Figure 1C), occlusion, variation in spike size, and partial spikes on the image edge (Yellow box with zoom-in shown in Figure 1D). The spike occlusion problem was the most serious problem and included various scenarios such as spikes over spikes (Figure 2A), leaves over spikes (Figure 2B), stems over spikes (Figure 2C), and awns over spikes (Figure 2D). Although the above factors greatly increase the segmentation difficulty, they encompass the true field environment and are helpful to improve the robustness of the spike segmentation model.

**FIGURE 3:**
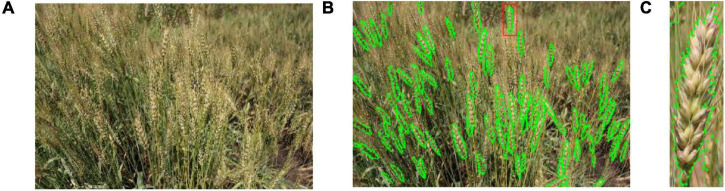
Annotation of wheat spikes: **(A)** the original image of a wheat plot, **(B)** the image with annotated wheat spikes, and **(C)** details of a single annotated spike.

The high complexity of the images brings great challenges to artificial annotation. The artificial image annotation software, Labelme (Russell et al., 2008), was used to label the ground truth for wheat spikes using polygons. Figure 3 shows the annotation of the images in this paper. To obtain high-quality annotated datasets, we enlarged the image about 200% or larger and selected the dense points along the outside edge of every spike to form an accurate spike region. However, there are still several very blurred spikes in the enlarged picture, which cannot be distinguished by the humans and are not annotated. Our group put a lot of effort in annotation and we believe this dataset can promote further wheat phenotypic studies. These annotated images were used to calculate the loss and optimize the model parameters during model training. In machine learning, about 2/3 to 4/5 of the datasets are usually used for training, and the remaining images are used for testing. Therefore, there are 524 images in the training set (12,591 spikes) and 166 images in the test set (4,934 spikes) in this paper.

### Methods of Wheat Instance Segmentation

#### Architecture of Wheat-Net

**FIGURE 4:**
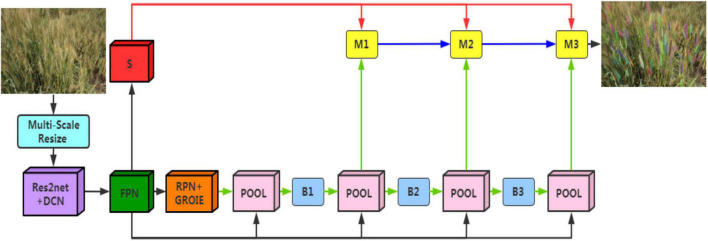
The architectures of Wheat-Net. “POOL” region-wise feature extraction, “B” bounding box, and “M” mask. “S” is semantic segmentation branch.

In this study, instance segmentation was the key protocol implemented to reliably detect and segment wheat spikes in a complex non-structural environment. We built the wheat spike instance segmentation model, Wheat-Net, for our high-complexity dataset based on the HTC model (Chen et al., 2019), which is a novel cascade architecture for instance segmentation. The HTC model has a powerful cascade structure that enhanced performance on various tasks. It solved the problem of insufficient information flow between mask branches at different stages in Cascade Mask RCNN, which is a direct combination of Cascade RCNN (Cai and Vasconcelos, 2021) and Mask RCNN (He et al., 2017). The HTC model effectively integrated cascade into instance segmentation by interweaving detection and segmentation for joint multi-stage processing, achieving outstanding performance on COCO (Common Objects in Context) test-dev and test-challenge (Lin et al., 2014). We cascaded three Mask RCNN networks to build the Wheat-Net (Figure 4). The advantages of this model can be ascribed to three key aspects. (1) It interleaved the box and mask branches (the green lines in Figure 4) based on Cascade Mask RCNN. This improvement allowed the mask branch to take advantage of the updated bbox. For instance segmentation of wheat spikes, the bbox information is very important for wheat mask segmentation. If bbox detects two adjacent spikes as the same object, the model will difficult to segment them. Therefore, the interleaving of box and mask branches can help to achieve more accurate wheat spike segmentation. (2) It made full use of the mask feature of the preceding stage by adding a direct information flow between mask branches (the blue lines in Figure 4). The direct information flow can learn more abundant multi-scale information of wheat from complex images, which further improved the accuracy of wheat segmentation. (3) It explored more contextual information by adding a semantic segmentation branch (the red lines in Figure 4), which can help the wheat spikes to be segmented accurately from the complex background. The above optimizations are combined (Equations 1–5) for better predictions, which effectively improved the utilization of information and enhanced performance.


(1) $${\text{r}}_{t}={\text{B}}_{t}\,\left({\text{x}}_{t}^{\mathrm{box}}\right)$$

(2) $${\text{x}}_{t}^{\mathrm{box}}=\text{p}\left(x,{\text{r}}_{t-1}\right)+\text{p}\left(\text{S}\left(x</cps:bf>\right),{\text{r}}_{t-1}\right)$$

(3) $${\text{x}}_{t}^{\mathrm{mask}}=\text{p}\,\left(x,{\text{r}}_{t}\right)+\text{p}\,\left(\text{S}\,\left(x\right),{\text{r}}_{t}\right)$$

(4) $${\text{m}}_{t}={\text{M}}_{t}\,\left(ℱ\,\left({\text{x}}_{t}^{\mathrm{mask}},{\text{m}}_{t-1}^{-}\right)\right)$$

(5) $$ℱ\,\left({\text{x}}_{t}^{\mathrm{mask}},{\text{m}}_{t-1}^{-}\right)={\text{x}}_{t}^{\mathrm{mask}}+{\text{g}}_{t}\,\left({\text{m}}_{t-1}^{-}\right)$$

Where x is the feature of the backbone network, $x_{t}^{\text{box}}$ and $x_{t}^{\text{mask}}$ denote box and mask features of *x* and the input Region of Interest (RoI). S indicates the semantic segmentation head. The box and mask heads of each stage take the RoI features extracted from the backbone as input. *p* (⋅) is a pooling operator, *B_t_* and *M_t_* indicate the box and mask head at the *t*-th stage.*r*_*t*_ and *m_t_* represent predictions of box and mask, respectively. $m_{t-1}^{-}$ indicates the intermediate feature of *M*_t–1_. ℱ is a function that combines the features of the current stage and the preceding one. *g_t_* denotes a 1 × 1 convolutional layer.

#### Optimization of Wheat-Net

**FIGURE 5:**
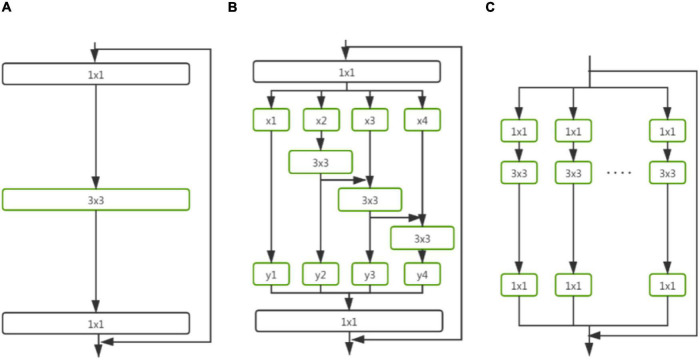
Comparison between different backbones for Wheat-Net. **(A–C)** Are the block of ResNet, Res2Net, and ResNeXt, respectively.

Different backbones have an important effect on the performance of the model because of their differences in feature extraction ability. Res2Net50 (Gao et al., 2021; Figure 5B) represents multi-scale features at a granular level and increases the range of receptive fields for each network layer, which is different from the concurrent bottleneck structure shown in Figure 5A, such as ResNet (He et al., 2016). Specifically, it replaces the 3 × 3 filters of n channels with a set of smaller filter groups, which are connected in a hierarchical residual-like style to increase the number of scales that the output features can represent. It can capture more details and global features of wheat without increasing calculations for wheat segmentation. ResNeXt (Xie et al., 2017) is an improved model of ResNet (Figure 5C), and is constructed by repeating a building block and the transformations to be aggregated, all of the same topology. It is a simple, homogeneous, and multi-branch architecture, which can extend to any large number of transformations without specialized designs. In the experimental part of this paper, we compare the performance of the above-mentioned backbones in our dataset.

**FIGURE 6:**
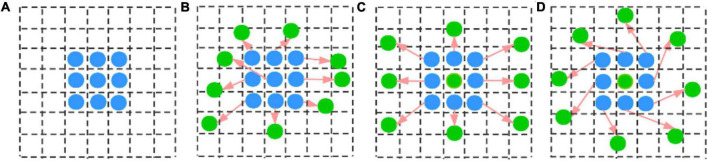
Different calculation positions under: **(A)** standard convolution (blue points); **(B)** deformable convolution (with green points). **(C,D)** Are special cases of **(B)**, showing that the deformable convolution generalizes scale and rotation transformations.

Deformable convolutional networks (DCN) (Dai et al., 2017) were integrated into our model because they provide a solution to model dense spatial transformations and are effective for sophisticated vision tasks. DCN allowed free deformation of the sampling grid as shown in Figure 6, which added offsets learned from target tasks to the regular sampling grid of standard convolution without additional supervision. DCN can help to solve the geometric deformation and enhance the robustness of the model for segmenting various sizes and angles of wheat spikes.

In our model, feature pyramid networks (FPN) (He et al., 2017) extracted RoI features from different levels of the feature pyramid by using a top-down architecture. These different features, generated and fused by FPN, comprised the inputs of the Region Proposal Network (RPN) (Ren et al., 2017). RPN predicted object bounds and objectness scores to efficiently generate region proposals with a wide range of scales and aspect ratios. Generic RoI Extractor (GRoIE) (Rossi et al., 2020) was used to extract the RoI. Since all layers of FPN retain useful information of wheat spikes, non-local building blocks and attention mechanisms were introduced to extract more information of wheat and overcome the limitations of existing RoI extractors, which select only one (the best) layer from FPN. They also can be integrated seamlessly with the two-stage architectures for instance segmentation tasks for superior performance compared to traditional RoI extractors (Pont-Tuset et al., 2017). Multi-task learning (Caruana, 1997) combined all tasks into a single model: that is, what is learned for each task can help other tasks be learned better. In this paper, we used multi-task learning to achieve both target detection and semantic segmentation of wheat spikes. Hence, Multi-task learning can improve learning efficiency and prediction accuracy by learning multiple objectives from a shared representation.

As an important part of the object detection pipeline, non-maximum suppression (NMS) could sort the detection bbox based on their scores (Rosenfeld and Thurston, 1971), select the detection bbox with the highest score and suppress all other bbox that had significant overlap (using a predefined threshold) with it. However, NMS might lose the objects that are within the predefined overlap threshold. Due to wheat is dense plant, there are a lot of overlap wheat spikes in the images. NMS might only detect one spike between two overlap spikes. Bodla et al. (2017) proposed a Soft-NMS algorithm to prevent objects from being eliminated. It decayed the detection scores of all other objects as a continuous function of their overlap. In the experimental part of this paper, we conducted a comparative experiment between NMS and Soft-NMS.

The size of the input image had a significant impact on model performance. Because we collected images with various shooting distances and angles, the dataset contained many small spikes. In our paper, small and big spikes are labeled as ground truth, which is more in line with the actual field of wheat. Because the feature map generated by the network was much smaller than the original image, the model may lose features of small spikes and unable to detect small spikes. Therefore, if the model fails to detect small wheat spikes, the performance of the model will be affected. Multi-scale training (He et al., 2015), which defines several fixed scales in advance and randomly selects a scale for training in each epoch, can effectively improve this limitation. Therefore, we used images of multiple scales for training to improve the robustness and accuracy of our model. Due to memory constraints, the short-side of the input images was randomly selected from 416 to 1,184, and another side’s size was calculated according to the aspect ratio of the original image’s size.

Learning rate (LR) was one of the most important hyperparameters in training. If the LR is large at the beginning of training, the model may become unstable, making it difficult to reach the optimal solution. To address this, we used warm-up LR (He et al., 2016) to improve the training situation. Warm-up LR allows the LR to gradually increase from a small value in the first few epochs until the initial LR is reached. In this way, the model can gradually stabilize, and the convergence speed becomes faster after stabilization.

As an important hyperparameter in deep learning, LR could determine whether and when the model can converge. A large LR will make the model fluctuate greatly, and it is difficult to reach the optimal solution. In addition, as the number of iterations increases, the LR will continue to decay to reduce fluctuations of model. We chose two popular LR decay methods and compared them in the experimental chapter: one was MultiStepLR, which used the dynamic step to update the LR, and the other was CosineAnnealingLR, which decayed the LR periodically based on the cosine function. Hyperparameters of the model were adjusted and optimized based on multiple experiments. Finally, the initial LR was set to 0.0025 and adjusted every 20 epochs with a decay factor of 0.5. The other hyperparameters of the model are shown in Table 1.

**TABLE 1:** Hyperparameter values which optimized through training.

Parameter	Value
Optimization algorithm	SGD
Momentum	0.9
Initial learning rate	0.0025
Warmup_iterations warmup_ratio = 0.001	500
Warmup_ratio	0.001
Optimal epoch	38
Batch size	1

Eventually, a new wheat spike segmentation method based on the HTC model combined with the backbone of Res2Net50, deformable convolutional networks, and Generic RoI Extractor was constructed (Figure 4). During the model training, each image was augmented using multiple methods (including VerticalFlip, RandomBrightnessContrast, RGBShift RGB, HueSaturationValue, ChannelShuffle, Blur, and MedianBlur) and the Res2Net50 backbone was pretrained based on the ImageNet dataset (Deng et al., 2009) using transfer learning, which was suitable for solving the problem of a small training dataset. The overall loss function takes the form of multi-task learning and was defined as Equation (6).


(6) $$\text{L}=\sum_{t=1}^{T}{\text{a}}_{t}\,\left({\text{L}}_{\mathrm{bbox}}^{t}+{\text{L}}_{\mathrm{mask}}^{t}\right)+\beta \,{\text{L}}_{\mathrm{seg}}$$

Where: $L_{\text{bbox}}^{t}$ is the loss of the bounding box predictions at stage t. $L_{\text{mask}}^{t}$ is the loss of mask prediction at stage t. *L*_seg_is the semantic segmentation loss in the form of cross-entropy. Because we cascade 3 Mask RCNN networks to build the Wheat-Net architectures, T was set to 3. In addition, to balance the contributions of different stages and tasks, we set α = [1, 0.5, 0.25] and β = 1 by default [31].

### Evaluation Metric

The performance of Wheat-Net was evaluated by average precision (AP), which is the area under the curve of precision-recall (PR) (Equations 7–9). A high AP value indicates that a model has both high precision and high recall. AP stood out as the most-used metric due to its representativeness and simplicity. AP was calculated (Equation 10) by using the method of the COCO dataset, which interpolated through all points. In this research, we evaluated the performance of Wheat-Net based on the IOU (Equation 11) threshold of 0.5, which is commonly used for instance segmentation model. The evaluation metrics are defined as follows:


(7) $$\text{Precision}=\frac{\text{TP}}{\text{TP}+\text{FP}}$$

(8) $$\text{Recall}=\frac{\text{TP}}{\text{TP}+\text{FN}}$$

(9) $${\text{p}}_{\mathrm{interp}}\,\left({\text{r}}_{n+1}\right)=\max_{\tilde{r}:\tilde{r}\geq r_{n+1}}\text{p}\,\left(\tilde{\text{r}}\right)$$

(10) $$\text{AP}=\sum_{n=0}\left({\text{r}}_{n+1}-{\text{r}}_{n}\right)\,{\text{p}}_{\mathrm{interp}}\,\left({\text{r}}_{n+1}\right)$$

(11) $$\text{IoU}\,\left(A,B\right)=\left|\frac{\text{A}\,⋂\text{B}}{\text{A}\,⋃\text{B}}\right|$$

Where TP indicates the correct detection of wheat spikes, FP is the wrong detection of wheat spikes, and FN represents the ground truth of wheat spikes not detected. Precision indicates how many wheat spikes detected by the model are real wheat spikes. Recall indicates how many real wheat spikes are detected by model in all real spikes. p (r^∼^) is the measured precision at recall (r.)^∼^ IOU is the intersection over union between two bboxes. A represents the bbox labeled manually and B represents the bbox generated based on Wheat-Net.

## Results

The data analysis was performed with the deep learning development framework of PyTorch. An Intel (R) Core (TM) i7-6700 processor, a 16GB random-access memory card, and a graphic card (NVIDIA GeForce GTX1080Ti 11GB) were used for the modeling process.

**FIGURE 7:**
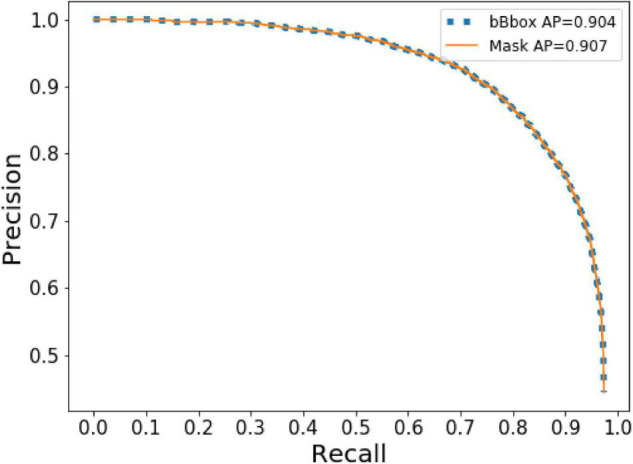
The curve of precision and recall.

To determine the appropriateness of the model, the test set was used to assess the model. The AP of bbox and mask reached 0.904 and 0.907, respectively. In the case of dense wheat spike detection from complex backgrounds, false positives tended to happen more often than false negatives. Therefore, we used the PR curve (Figure 7), which emphasized the evaluation of the prediction model on positive examples to evaluate the performance of the model. This step confirmed the effectiveness of Wheat-Net for detecting wheat spikes in the complex field environment.

**FIGURE 8:**
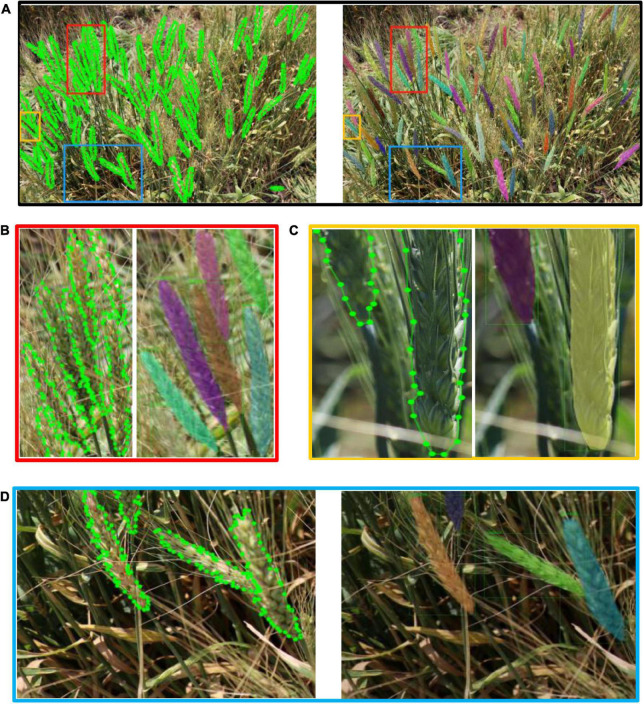
Annotation images vs. detection results. **(A)** Overall detection results, **(B)** detail 1—area of adjacence and occlusion, **(C)** detail 2—area of incomplete spikes in image, **(D)** detail 3—area of insufficient illumination of spikes.

In addition, we visualized the detection results of the complex image shown in Figure 8A. As shown in Figure 8, in the non-structural field, the model showed outstanding performance for complex backgrounds, dense spikes, adjacency, and occlusion (Figure 8B), insufficient illumination (Figure 8D), and incomplete spikes on the edge of images (Figure 8C).

**FIGURE 9:**
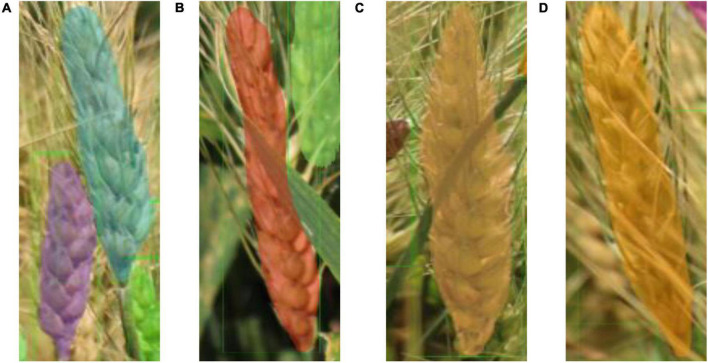
Detection results of various occlusions. **(A)** One spike (under purple mask) is occluded by another spike, **(B)** one spike (under red mask) is occluded by a wheat leaf, **(C)** one spike is occluded by a wheat stem, **(D)** One spike (under orange mask) is occluded by awns from another spike.

The model can effectively solve the problem of various occlusion scenarios, which is one of the most challenging areas in the field of object detection. Figure 9 demonstrated the detection results of various occlusion scenarios including: when a spike is obstructed by another spike; when a spike is occluded by a leaf; when a spike is occluded by a stem; and when a spike is occluded by awns from another spike. Comparing the total number of spikes (4,899) detected by the model with the actual number of spikes manually labeled (4,934), 99.29% of the manually labeled wheat spikes (clearly visible to humans) are detected. It should be noted that the main goal of this paper is to accurately segment wheat spikes in complex environments, so the datasets and scenarios may different from pure wheat counting studies. This demonstrates that Wheat-Net was effective for automatic wheat spike detection under complex field conditions. In addition, the instant segmentation algorithm is used to segment wheat spikes out of plot images. Segmentation provides information such as size, shape, and relative location of the segments in the image, which can be used for phenotypic traits such as spike size, shape, distribution, and wheat yield potential.

### Ablation Study

Ablation study is an effective way to see how a method affects the performance of the entire model by removing that specific method from the model. To perform this analysis, we used multi-scale training, DCN, and GRoIE methods to improve the performance of the model. To more accurately evaluate the effect of each method, we conducted the ablation study experiments with the Wheat-Net based on Res2net50 (LR = 0.0025, batch size = 1, image scale = 2,100*1,184) and compared the performances on the test set.

The experimental results (Table 2) showed that multi-scale training, DCN, and GRoIE had various effects on the performance of Wheat-Net. Specifically, the AP (both IOU = 0.5 and IOU = 0.75) were significantly improved by multi-scale training, although it increased some test times. The improvement of DCN for IOU = 0.75 was greater than that for IOU = 0.5, which showed that DCN had a more significant effect on a large IOU threshold. In addition, GRoIE increased AP with IOU = 0.5 and decreased AP with IOU = 0.75. The experimental results showed that GRoIE did not work for our dataset when using a larger threshold of IOU.

**TABLE 2:** The results of ablation study.

Multi-scale	DCN	GRoIE	AP (IOU = 0.5)		AP (IOU = 0.75)		Epoch	Train time/h	Test time/s
			Bbox	Mask	Bbox	Mask			
**−**	**−**	**−**	0.868	0.872	0.722	0.677	20	7.5	2,988
**√**	**−**	**−**	0.891	0.899	0.772	0.745	40	14	4,775
**√**	**√**	**−**	0.897	0.904	0.794	0.768	40	14	4,791
**√**	**√**	**√**	0.904	0.907	0.790	0.747	38	16	2,560

### Comparative Evaluation

To make full use of the advantages of the Wheat-Net to achieve better performance, we conducted experiments to select the best backbone to build the Wheat-Net. As shown in Table 3, we selected ResNet50, ResNet101, ResNeXt50, ResNeXt101, and Res2Net50 for comparative experiments. By comparing the results of ResNet50 and ResNet101 (or ResNeXt50 and ResNeXt101), we found that increasing the depth of the backbone could not improve the performance of wheat spike detection. In general, the more layers the deep neural network has, the stronger the fitting ability of the model will be. In practice, there is not only ground truth but also noise in the image. The stronger the fitting ability of the model, the stronger the ability to learn noise. In particular, the noise in this paper (such as blurry spikes, leaf, stem, and awns) is similar to the ground truth in color and texture, which makes it more difficult for the model to distinguish between noise and ground truth. Therefore, in the situation of this paper, just increasing the depth of the backbone may not represent a better effect. The test results (Table 3) showed that Res2Net50 and ResNeXt50 have more outstanding performance than other backbone networks. Furthermore, ResNeXt50 required a shorter test time, while Res2Net50 had higher AP values. Both of the two networks can be used as the backbone of our model based on different criteria. In this paper, based on the requirement of precision, we chose Res2Net50 as the backbone network of Wheat-Net for segmenting wheat spikes in the complex field. In practical applications, if there is a higher requirement for the model speed, ResNeXt50 will be suitable to be the backbone network.

**TABLE 3:** Comparative experimental results.

Type	AP (IOU = 0.5)		AP (IOU = 0.75)		Epoch	Train time/h	Test time/s
	Bbox	Mask	Bbox	Mask			
**ResNet50**	0.894	0.897	0.761	0.693	60	17	2,393
**ResNet101**	0.871	0.876	0.696	0.632	20	7	3,302
**ResNeXt50**	0.893	0.897	0.788	0.733	66	18	2,299
**ResNeXt101**	0.872	0.875	0.706	0.624	40	14	2,251
**Res2Net50**	0.904	0.907	0.790	0.747	38	16	2,560
**NMS**	0.904	0.907	0.790	0.747	38	16	2,560
**Soft-NMS**	0.903	0.906	0.795	0.750	38	16	3,385
**CosineAnnealingLR**	0.891	0.895	0.786	0.753	70	18	3,134
**MultiStepLR**	0.904	0.907	0.790	0.747	38	16	2,560
**Wheat-Net**	0.904	0.907	0.790	0.747	38	16	2,560
**Optimized mask RCNN**	0.884	0.884	0.755	0.690	60	10	3,186
**Optimized cascade mask RCNN**	0.899	0.900	0.785	0.754	40	14	2,673

In object detection, the model will generate a lot of region proposals, and the suppression algorithm is needed to remove redundant region proposals to reduce the number of parameters in the model. In this paper, we conducted a comparative experiment between NMS and Soft-NMS to achieve the better performance of Wheat-Net. As shown in Table 3, the Wheat-Net with NMS achieved a higher AP with IOU = 0.5 within a much shorter test time compared to Soft-NMS. Therefore, although Soft-NMS could help the Wheat-Net to achieve slightly higher AP with IOU = 0.75, we chose to use NMS based on the best balance between precision and speed.

In the training process, if the LR is too large, the model will be difficult to converge, if the LR is too small, the convergence speed will be slow. Therefore, the dynamic decay of LR is extremely important to make the model faster and more stable to convergence. In order to choose a more suitable method of LR decay, we conducted a comparative experiment between MultiStepLR and CosineAnnealingLR to select the suitable LR decay method for Wheat-Net. From Table 3, we can see that the MultiStepLR was superior to CosineAnnealingLR in terms of AP. In addition, MultiStepLR converged faster and required a shorter test time than CosineAnnealingLR. Therefore, MultiStepLR was better than CosineAnnealingLR in terms of accuracy and speed for our model of wheat spike detection, so we chose MultiStepLR to decay the learning rate and further improve the performance of our model.

In order to evaluate the advantages of the hybrid cascade structure of Wheat-Net, we first used the same optimization method (including multi-scale training, DCN, and GRoIE) to optimize the Mask RCNN and Cascade Mask RCNN, and then conducted a comparative experiment. As shown in Table 3, we can seen that the box AP and mask AP of the Wheat-Net are better than the other two models. In addition, although the train time of the Wheat-Net was slightly longer, the Wheat-Net was more satisfactory in terms of test time and converged in the lowest number of epochs. The above analysis proves that the hybrid cascade structure of Wheat-Net is very effective for segmenting wheat spikes in the field environment.

## Discussion

### Analysis of Experimental Error

**FIGURE 10:**
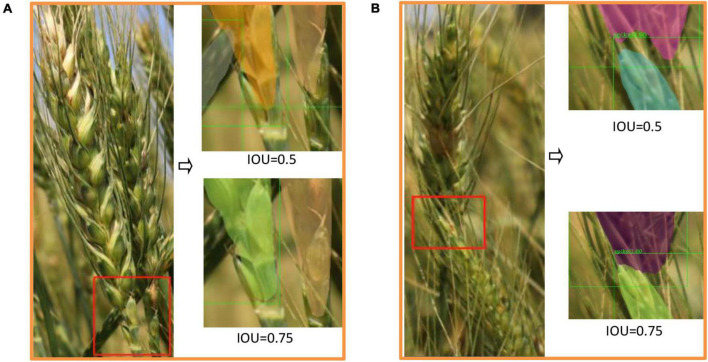
The Demonstration of experimental error. **(A)** The error of spike bottom. **(B)** The error at the junction of different spikes.

Although Wheat-Net showed excellent performance for wheat segmentation in the complex environment, there were still errors, which we subsequently analyzed. As shown in Figure 10A, the model had some segmentation errors at the bottom of the wheat spikes. The sparse florets at the bottom of the spike led to some differences between the texture characteristics of the bottom and other parts. This in turn caused inaccurate segmentation for the bottom of the spikes.

Due to the complexity of our dataset, the problem of adjacence and occlusion of wheat was very common in most images. Segmentation of adjacent objects was one of the most challenging tasks in the field of crop phenotyping. From Figure 10B, we can see that the two spikes were adhesive and the lower one was occluded by a wheat stem. Our method achieved a good segmentation result in such a complex situation, but there were still errors at the junction. The color, texture, and shape of the adherent spikes were very similar, which made the dividing line unclear. As a result, this made the positive objects at the junction annotated as negative, which increased the number of False Negatives (FN) and reduced Recall. When multi-scale training and DCN were used (GROIE was not used), the AP value with IOU of 0.75 was the highest (Table 3). We chose this model for visual testing (shown in Figure 10) and found that it could greatly reduce the above-mentioned experimental errors (including the bottom and junction errors) compared to when IOU = 0.5. Regardless of the ability to detect wheat spikes, it achieved better performance for accurately segmenting the wheat spike.

### Evaluation of Wheat-Net on Barley Spike Detection

**FIGURE 11:**
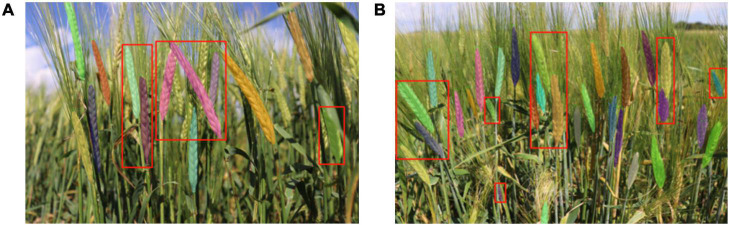
The visualization results of barley detection. **(A,B)** The two examples of barley detection.

The phenotypic characteristics of wheat and barley are quite different in both the shape and size of the kernel and the length of the awn. In order to verify the generalized applicability of the model, we constructed a test set containing 29 barley images to test the detection ability of the model to barley spikes. The experimental results showed that the AP of bbox and mask for barley detection achieved 0.799 and 0.812, respectively. From Figure 11, we can see that the model achieved acceptable visualization results for barley, especially for the detection of adjacence and occlusion (red boxes in Figure 11). Thus, our model has the potential to segment barley spikes as well demonstrating strong robustness to a variety of spike shapes and colors. However, due to the similar phenotypic characteristics of adhesive spikes, there were still errors at the junction of spikes. In addition, similar to the errors encountered with wheat spike detection, there were also some errors at the top and bottom of barley spikes. It is expected that the performance of segmenting barley spikes will be improved by retraining our model using a barley training dataset. This study also established the protocol of a pretraining model for the detection of other inflorescences of small grain cereal crops such as the panicles of oat and rice.

### Comparison of Wheat Detection Methods

Compared with the conventional wheat detection methods (Alkhudaydi et al., 2019; Qiu et al., 2019; Zhang et al., 2019; Ma et al., 2020; Tan et al., 2020; Su et al., 2021), the proposed Wheat-Net in this paper showed a preferable performance for instance segmentation in various complex scenes, including complex backgrounds, insufficient illumination, dense wheat spikes, spike adjacency, and occlusion. In our previous research (Su et al., 2021), we basically realized the instance segmentation of wheat in a complex field by using only a single Mask RCNN. However, since the method with a single Mask RCNN cannot learn sufficient features from our complex datasets, it had a poor effect (especially for segmentation of partial spikes of the image edge and occlusive spikes). Therefore, we spent a year devoted to improving the performance of previous study. Eventually, compare with only a single Mask RCNN of Su et al. (2021), we cascaded three Mask RCNN to construct the Wheat-Net of hybrid cascade structure, and with Res2Net50 as the backbone network, multi-scale training, DCN, and GRoIE were used to learn abundant features of different scales. From the bold values in Table 4, we can see that the AP of bbox is increased by 0.337 (from 0.567 to 0.904), and the AP of mask is increased by 0.335 (from 0.572 to 0.907). In addition, we can see from Figures 8, 9 that Wheat-Net achieved excellent performance for partial spikes of the image edge and occlusive spikes, which had a poor effect on the method of Su et al. (2021). The above experiment shows that our method can overcome various challenges in the complex field and achieve accurate and efficient instance segmentation of wheat spikes.

**TABLE 4:** Comparison of the AP of Wheat-Net vs. other models in wheat detection.

Model	AP (IOU = 0.5)	

	Bbox	Mask
Su et al. (2021)	0.567	0.572
**Wheat-Net**	0.904 (**↑0.337**)	0.907 (**↑0.335**)

## Conclusion

Due to the high complex dataset (complex backgrounds, serious occlusion), an effective instance segmentation method based on the HTC model was established to automatically segment wheat spikes in the fields. The proposed method with a hybrid cascade structure to make full use of rich mask and box information. With Res2Net50 as the backbone network, multi-scale training was used to learn features of different scales, and deformable convolutional networks (DCN) and Generic RoI Extractor (GRoIE) were trained to improve model accuracy. Based on the methodology, the difficulties of complex backgrounds, serious occlusion, and incomplete spikes on the edge were solved with AP of 0.904 and 0.907 for bbox and mask, respectively. The accuracy rate for wheat spike counting was 99.29%. Comprehensive empirical analyses revealed that the proposed method was particularly effective for the detection of wheat spikes with frequent adjacence, overlapping, occlusion, and other complex growth states. This study achieved excellent performance for dense wheat spike segmentation with complex field, which is conducive to promoting production and management of wheat. However, field data collection is limited to only the crop season, which is 3 months per year in Minnesota. One solution is to expand the data collection window by conducting multi-site data collection across regions. In addition, we will study the method of data augmentation based on the Generative Adversarial Network (GAN). Our models will also be used in the large-scale wheat field trials. We expect that our proposed method will be expanded to the broader agricultural research area, including detection of the seed-bearing inflorescences of other crops.

## Data Availability Statement

The raw data supporting the conclusions of this article will be made available by the authors, without undue reservation.

## Author Contributions

JZ, AM, CY, and BS: conceptualization. JZ, AM, QM, and CY: methodology and formal analysis. JZ, AM, CY, and JW: software. JZ, QM, AM, CY, and JW: validation. CY, CH, W-HS, and BS: investigation. JZ, AM, CY, CH, JA, and BS: data curation. JZ: writing-original draft preparation. JZ, AM, CY, QM, BS, CH, and JA: writing-review and editing. CY, BS, W-HS, and QM: project administration. All authors have read and agreed to the published version of the manuscript.

## Conflict of Interest

The authors declare that the research was conducted in the absence of any commercial or financial relationships that could be construed as a potential conflict of interest.

## Publisher’s Note

All claims expressed in this article are solely those of the authors and do not necessarily represent those of their affiliated organizations, or those of the publisher, the editors and the reviewers. Any product that may be evaluated in this article, or claim that may be made by its manufacturer, is not guaranteed or endorsed by the publisher.
